# Repeated unilateral handgrip contractions alter functional connectivity and improve contralateral limb response times

**DOI:** 10.1038/s41598-023-33106-1

**Published:** 2023-04-20

**Authors:** Justin W. Andrushko, Jacob M. Levenstein, Catharina Zich, Evan C. Edmond, Jon Campbell, William T. Clarke, Uzay Emir, Jonathan P. Farthing, Charlotte J. Stagg

**Affiliations:** 1 College of Kinesiology, University of Saskatchewan, Saskatoon, Canada; 2 FMRIB, Nuffield Department of Clinical Neurosciences, Wellcome Centre for Integrative Neuroimaging, University of Oxford, Oxford, UK; 3 Section on Functional Imaging Methods, National Institutes of Mental Health, National Institutes of Health, Bethesda, MD USA; 4 Thompson Institute, University of the Sunshine Coast, Sippy Downs, Australia; 5 Department of Clinical and Movement Neurosciences, University College London, London, UK; 6 School of Health Sciences, College of Health and Human Sciences, Purdue University, West Lafayette, USA

**Keywords:** Neuroscience, Motor control, Sensorimotor processing

## Abstract

In humans, motor learning is underpinned by changes in sensorimotor network functional connectivity (FC). Unilateral contractions increase FC in the ipsilateral primary motor cortex (M1) and supplementary motor area (SMA); areas involved in motor planning and execution of the contralateral hand. Therefore, unilateral contractions are a promising approach to augment motor performance in the contralateral hand. In a within-participant, randomized, cross-over design, 15 right-handed adults had two magnetic resonance imaging (MRI) sessions, where functional-MRI and MR-Spectroscopic Imaging were acquired before and after repeated right-hand contractions at either 5% or 50% maximum voluntary contraction (MVC). Before and after scanning, response times (RTs) were determined in both hands. Nine minutes of 50% MVC contractions resulted in decreased handgrip force in the contracting hand, and decreased RTs and increased handgrip force in the contralateral hand. This improved motor performance in the contralateral hand was supported by significant neural changes: increased FC between SMA-SMA and increased FC between right M1 and right Orbitofrontal Cortex. At a neurochemical level, the degree of GABA decline in left M1, left and right SMA correlated with subsequent behavioural improvements in the left-hand. These results support the use of repeated handgrip contractions as a potential modality for improving motor performance in the contralateral hand.

## Introduction

The acquisition of motor skill is essential in our daily lives. Able-bodied individuals may take for granted the abilities to grasp and manipulate objects, or even to safely complete essential activities of daily living. However, many people lose their ability to perform these tasks through a variety of injuries. Restoring function in these individuals is of clear clinical importance, but *how* we can optimally improve behaviour is an open scientific question: both in terms of restoring motor function in orthopedic or neurologically impaired individuals such as stroke survivors, and in healthy populations, such as athletes, looking to maximize motor performance.

At a systems level, studies suggest that motor performance can be enhanced through interventions that either increase corticomotor excitability^1–4^ and/or decrease corticomotor inhibition. Gamma-aminobutyric acid (GABA)-ergic inhibition is shown to decrease during motor learning^5,6^, and the magnitude of this GABA decrease correlates with the extent of subsequent motor performance improvement^7^. Altering motor cortex (M1) excitability and inhibition through non-invasive brain stimulation techniques increases motor skill acquisition, both in neurologically intact adults^8,9^ and in functional recovery after stroke^10,11^.

However, non-invasive brain stimulation is not widely available, and determining other methods to alter corticomotor function is an important research objective. One approach that is widely accessible and has shown promise for altering the cortical excitation/inhibition balance is performing a unilateral exercise protocol that induces motor fatigue. At a neurochemical level, unilateral fatiguing exercise decreases M1 GABA_A_ activity, as quantified by the Transcranial Magnetic Stimulation protocol Short-interval Intracortical Inhibition (SICI) in the contralateral M1 (cM1)^12^ and ipsilateral M1 (iM1)^13^. Unilateral fatiguing exercise also increases iM1 cortical excitability^14,15^, and functional connectivity in the sensorimotor network, particularly in the iM1, ipsilateral supplementary motor area (SMA), and contralateral SMA^16^. Unilateral fatiguing exercise has been shown to enhance muscle activity in the contralateral unfatigued homologous muscle^17^, although this finding is not consistent across the literature^18,19^. Recent work has shed light on the role of the non-decussating cortico-reticulospinal tract for modulating upper limb voluntary motor control^20–24^. Specifically, excitability of this pathway is known to reduce response times^24^, and this pathway is also linked to enhanced neuromuscular strength^22,25^. As this pathway has origins in the SMA^26^ we wished to understand whether these ipsilateral cortical alterations in M1 and SMA serve as a neural basis for contralateral behavioural improvements in neuromuscular strength via a handgrip strength task and response times using a visually cued response time task.

**Figure 1:**
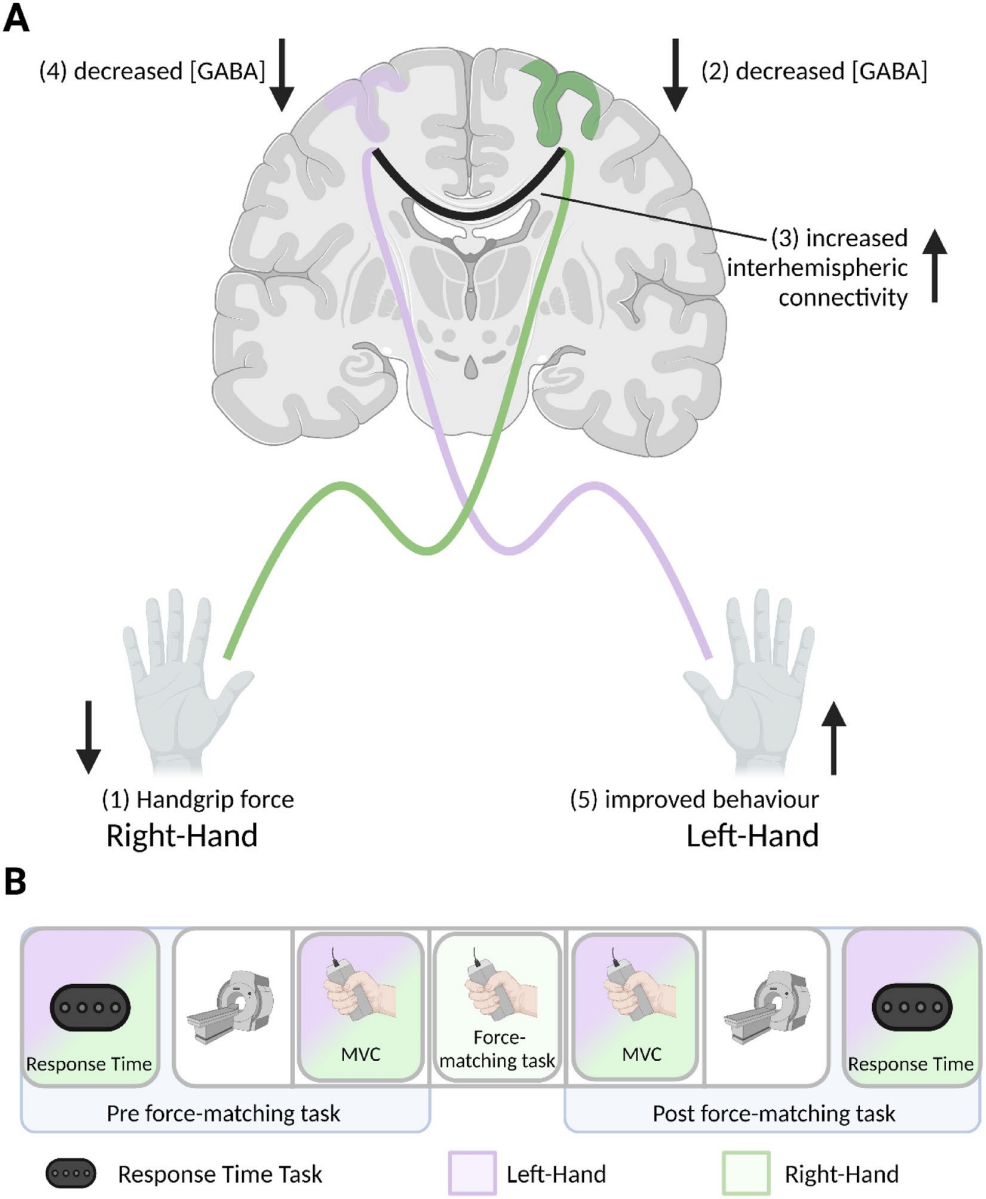
(**A**) Theoretical physiological model for the present study, (**B**) Schematic outlining the study design. MVC = maximum voluntary contraction; Force-matching task = nine-minutes of 0.5 Hz right handgrip contractions performed at either 5% or 50% MVC. This figure was created with BioRender.com.

Considering motor network connectivity is at least in part controlled by M1 inhibition^27,28^, we acquired two independent measures [resting-state functional magnetic resonance imaging (rs-fMRI), and resting-state magnetic resonance spectroscopic imaging (rs-MRSI)] to test the hypothesis that repeated unilateral handgrip contractions, resulting in improved performance in the opposite hand (i.e., faster response times and enhanced handgrip strength) are related to increased interhemispheric homologous connectivity of M1, and SMA via increased glutamate and/or decreased GABA in these regions (Fig. 1A).

## Methods

This study conforms to the Declaration of Helsinki and was approved by the Oxford Central University Research Ethics Committee (MSD-IDREC-C1-2014-100 and MSD-IDREC-C1-2014-090). Using an effect of η^2^ = 0.250 based on the relevant interaction from a previous brain stimulation study (transcranial direct current stimulation (anodal, cathodal, bilateral sham) × neurochemical (GABA, glutamate) × hemisphere (Left M1, right M1) × time (during stimulation, post 1, post 2, post 3))^29^, we computed an estimated total sample size of 12 (G*Power 3.1.9.2; 1-β = 0.95, α = 0.05). This higher-order interaction was chosen given the similarity in study design of a unilateral protocol designed to induce interhemispheric interactions at a neurochemical level. To allow for dropout and data loss, we therefore recruited fifteen right-handed participants [Edinburg Handedness Questionnaire – short form (EHQ)^30,31^] aged between 18 and 35 years (28.9 ± 3.3 years; 7 Female; EHQ: 85.8 ± 18.8). Exclusion criteria were: playing a musical instrument to Associated Board of the Royal Schools of Music Grade 5 or higher, any contraindication to MRI, any history of neurological or psychiatric disorders. Written informed consent was obtained from all participants prior to their participation in the present study.

### Experimental outline

Participants had two experimental sessions where they performed a right handgrip force-matching task at either 5% or 50% of their maximum voluntary contraction (MVC) in a randomized order (n = 7 started with 5% MVC; n = 8 started with 50% MVC). During each experimental session, each hand’s response times (RTs) were assessed before and after the participant entered the MRI. Inside the scanner, resting-state functional MRI (fMRI), magnetic resonance spectroscopic imaging (MRSI), and three unilateral MVCs with each hand were acquired before and after a nine-minute right handgrip force-matching task at either 5% or 50% MVC was performed. This study had a within-participant repeated measures design, with the order of conditions stratified-randomized for sex across the group (Fig. 1B).

### Handgrip force-matching task

The handgrip force-matching task was performed using an MRI-compatible hand dynamometer (Biopac Systems Inc. Aero Camino Goleta, CA) and implemented using in-house code (Psychtoolbox-3^32,33^). Participants performed a visually cued 0.5 Hz (1 s contraction, 1 s rest) repeated force-matching task at either 5% or 50% of their MVC, depending on the session. The MVC of each hand was tested immediately before and after executing the submaximal handgrip task in the MRI. The target force was displayed halfway up the vertical axis, and participants were instructed to squeeze the dynamometer to guide a cursor up the screen until it reached the target. While in the MRI, participants performed 270 handgrip contractions over the nine-minute task.

#### Handgrip force-matching task analysis

The area under the curve (AUC) of the contraction force was calculated over a two second window for every contraction separately. A regression line was then fitted to the AUC measures across all contractions for each participant and session separately. The β of the regression from each session was used to quantify motor performance, whereby a β < 0 indicates a decrement in motor performance over time, consistent with fatigue.

### Response time task

Participants performed a visually-cued response time task before and after MRI scanning, implemented in PsychoPy3^34^*.* Participants responded via a button box (4-Button Inline, HHSC-1 × 4-L; Current Designs Inc. Philadelphia, PA USA) with their right or left hand depending on the block. The task was divided into blocks of 64 self-paced visual cues on a computer monitor in a random order, which participants were asked to respond to by pressing the corresponding button on the button box as quickly and accurately as possible. With each hand separately, participants performed two blocks at the pre force-matching task and post force-matching task timepoints (Fig. 1B).

#### Response time task analysis

Incorrect button-press responses, and response times (RTs) < 50 ms or > 700 ms were removed (a total of 656 correct presses were removed across all participants and sessions). This approach to exclude invalid RTs is similar to previous literature^7,35,36^. The median response time of the remaining button presses was then extrapolated for each block separately. To investigate changes in RT due to repeated handgrip contractions, the percent change (percent change = post – pre/pre * 100) in RTs for each hand was calculated using the mean of the medians from the two pre force-matching task blocks as the pre force-matching task value, and similarly, the mean of the medians from the two post force-matching task blocks was used as the post force-matching task value. To investigate any significant change in RT between pre and post force-matching task, we performed a repeated measures analysis of variance (RM-ANOVA) using the percent change in RT data with one factor of condition (5% MVC, 50% MVC) and another of hand (Left-hand, right-hand).

### Magnetic resonance imaging

MRI sessions were acquired using a Siemens 3-T Prisma whole-body MRI scanner with a 32-channel head array receive coil (Siemens, Erlangen, Germany). T1-weighted MPRAGE scans [voxel size 1 mm^3^, TR = 1900 ms, TI = 912 ms TE = 3.96 ms, FOV = 232 × 256 × 192 mm^3^, flip angle = 8°, total acquisition time = 7:21 (minutes:seconds)] were acquired for registration purposes and to guide placement of the MRSI slab. An example of the MRSI slab placement can be seen in Fig. 4A. The slab was placed manually in each session in order to capture bilateral motor (hand area) and premotor areas while prioritising left M1 and avoid non-brain tissue. The slab was therefore placed as superiorly, then laterally, and then anteriorly as possible, while ensuring good coverage of left M1 and no inclusion of dura. After initial slab placement screenshots were taken and used as a reference to replicate the same slab location across timepoints and sessions. Pre- and post force-matching task resting-state semi-LASER localised density-weighted concentric-ring trajectory MRSI (voxel size 5 mm × 5 mm × 15 mm, semi-LASER VoI 85 mm × 35 mm × 15 mm, TR = 1400 ms, TE = 32 ms, FOV = 240 mm × 240 mm × 15 mm, total acquisition time = 4:30)^37^, and fMRI (490 volumes, voxel size 2.4 mm isotropic with zero gap, TR = 735 ms, TE = 39 ms, FOV = 210 × 210 × 154 mm, flip angle = 52°, total acquisition time = 6:10)^38^ data were acquired. Participants were asked to fixate on a grey cross presented on a black screen and to blink normally. To quantify any potential change in head position after task performance a single localiser (voxel size 1.4 mm, TR = 3.15 ms, TE = 1.37 ms, FOV = 350 mm × 350 mm × 263 mm, 128 slices, flip angle = 8°, total acquisition time = 0:22) was collected prior to Post-task MRSI. Finally, B_0_ field maps (voxel size 2 mm^3^, TR = 482 ms, TE1 = 4.92 ms, TE2 = 7.38 ms, FOV = 216 mm × 216 mm × 146 mm, 49 slices, flip angle = 46°, total acquisition time = 1:45) were acquired to correct for field distortion in the resting-state fMRI data. The entire scan duration including the nine-minute handgrip force-matching task was approximately 40 min per session.

### MRI analysis

#### fMRI preprocessing

Functional MRI analyses were performed using tools from the FMRIB Software Library (FSL v6.0.3^39^). Standard preprocessing steps were performed, including removal of non-brain tissue (BET^40^), removal of the initial two volumes, motion correction (MCFLIRT^41^), high-pass temporal filtering at 0.01 Hz, and distortion correction with the implementation of field-maps. For motion parameter data see supplementary information.

Following single-participant MELODIC preprocessing (v3.15), FMRIB's Independent Component Analysis (ICA)-based Xnoiseifier (ICA-FIX) was used to automatically denoise the data^42,43^. The UK Biobank training-weights file (UKBiobank.RData) was used with a threshold value of 20, and 0.01 Hz high-pass filtered motion confound cleanup. Following the automated denoising, all components were manually inspected before the cleaned data were smoothed with a 5 mm full-width half maximum (FWHM) kernel.

Individual resting state fMRI scans were first registered to the respective T1 structural scan using boundary-based registration as implemented in FMRIB's Linear Image Registration Tool (FLIRT^41,44^), and then to a standard space template (MNI152 2 mm) using non-linear registration (FNIRT^45,46^).

#### fMRI analysis

We assessed resting state functional connectivity using two different approaches: (1) a seed-based functional connectivity approach to test the hypothesis that unimanual handgrip contractions lead to changes in connectivity of the ipsilateral right M1 (rM1); and (2) a region of interest (ROI)-based functional connectivity analysis to specifically address our a priori hypothesis that unimanual handgrip contractions would increase M1–M1 and SMA–SMA connectivity.

1. *Seed-based, whole-brain functional connectivity*

The hand area of the rM1 (corresponding to non-contracting left hand) was defined from previous functional MRI data of hand movements^47^, and the mean timeseries was extracted from this region for each participant and each session independently. This was then entered as a regressor into a lower-level FEAT analysis^48^, and task-related changes in functional connectivity of rM1 were investigated via a higher level mixed-effects analysis using a cluster forming threshold of z = 3.1 and *p *= 0.05^49^.

(2) *ROI-ROI-based functional connectivity*

Next, we wanted to specifically assess changes in M1–M1 and SMA–SMA functional connectivity. The hand area of the M1s was functionally defined as above, and SMA was defined from a previous study that created an MNI template tractography-based parcellation that had excellent spatial correlations with SMA activation measured with fMRI during a sequenced finger tapping task that is known to evoke SMA activity^50^. The mean timeseries from within each region was extracted for each participant and session separately. We calculated the Pearson’s r values between the homologous pairs using custom in-house MATLAB scripts, which were then converted to z-scores using a Fishers *r* to z transformation. The z-scores for the correlation between the homologous pairs were then used in a 2 × 2 × 2 [condition (5%, 50% MVC) × ROI (M1, SMA) × time (pre force-matching task, post force-matching task)] RM-ANOVA.

### MRSI

MRSI data was reconstructed and preprocessed according to Steel et al.^37^, using in-house scripts. Reconstruction and preprocessing included: (i) metabolite cycling reconstruction^51^, (ii) coil-combination^52^, (iii) frequency and (iv) phase shift correction, (v) HLSVD for residual water removal^53^, and (vi) eddy current correction using the unsuppressed water signal^54^. Concentration of neurochemicals was quantified as in Steel et al.^37^ using LCModel^55,56^. A chemical shift of 0.5 to 4.2 ppm was evaluated with a basis set containing 20 metabolites and default LCModel macromolecules with disabled soft constraints on metabolites (NRATIO set to 0), and a baseline stiffness setting (DKMTM) of 0.25 (see Fig. 4A for example of raw spectra and model fits). Each voxel spectrum was independently fit with spectral quality and assessment of model fitting performed using in-house scripts. For the metabolite maps of glutamate + glutamine (Glx) and GABA, MRSI voxels with Cramer-Rao lower bound values > 50 or signal to noise ratio < 40 were excluded from further analysis. All metabolite measurements are expressed as a ratio over total Creatine (tCr).

Metabolite maps were next upsampled to 1mm^3^ resolution using nearest neighbour interpolation which preserves the original sampling grid. Metabolite maps were then aligned to their native T1 coordinate space. To correct for potential shift in head position, structural scans acquired prior to each MRSI sequence were aligned using MCFLIRT^41^. The metabolite maps were then transformed using the MCFLIRT generated registration matrix. Only shared MRSI voxels from within an intersection mask of QA passed and head position aligned MRSI voxels across each timepoint were included in subsequent analyses. For the ROI analysis, the four binary ROI masks [left M1 (lM1), right (rM1), left SMA (lSMA) and right SMA (rSMA)] were non-linearly aligned to native T1 space by inverse warping (3dNwarpApply) the native to standard space alignment, generated with @SSwarper^57–59^. The native space ROIs were thresholded at 0.5 to mitigate partial volume effects. Within the intersection mask, mean concentration of Glx/tCr and GABA/tCr were then extracted for each of the four ROIs.

### Statistical analyses

Statistical analyses were run using Jamovi v1.6.9^60^. An alpha level for significance testing was set to 0.05. Hedges’ g effect sizes are reported for t-tests and partial eta squared (η_p_^2^) effect sizes are reported for ANOVA results. Greenhouse–Geisser corrections were applied as necessary where violations of sphericity were present. Shapiro–Wilk tests were used to assess data normality. Additionally, the order of sessions was first included in each of the RM-ANOVA models as a covariate, but after determining that the order of testing was not a significant covariate in each of the models (i.e., it did not significantly adjust the dependent variables due to between group differences) it was not included in the final analyses. For MRI analyses that report change scores, these were calculated by subtracting the pre force-matching task from the post force-matching task MRI data.

### Significance statement

Enhanced functional connectivity and decreased inhibition in sensorimotor areas of the brain underpin enhancements in motor performance and learning. In this study we investigated the impact of repeated right handgrip contractions at 50% MVC to improve behaviour, enhance functional connectivity and alter sensorimotor inhibition. We found that after nine minutes of repeated 50% MVC handgrip contractions with the right-hand, left-hand response times and handgrip force were significantly improved. This behavioural improvement was accompanied by altered interhemispheric functional connectivity and neurochemical changes across sensorimotor areas. Repeated unilateral handgrip contractions may be an effective method for enhancing contralateral limb motor performance in rehabilitation settings.

## Results

### Performance on the 50% MVC handgrip force-matching task decreased over time

**Figure 2:**
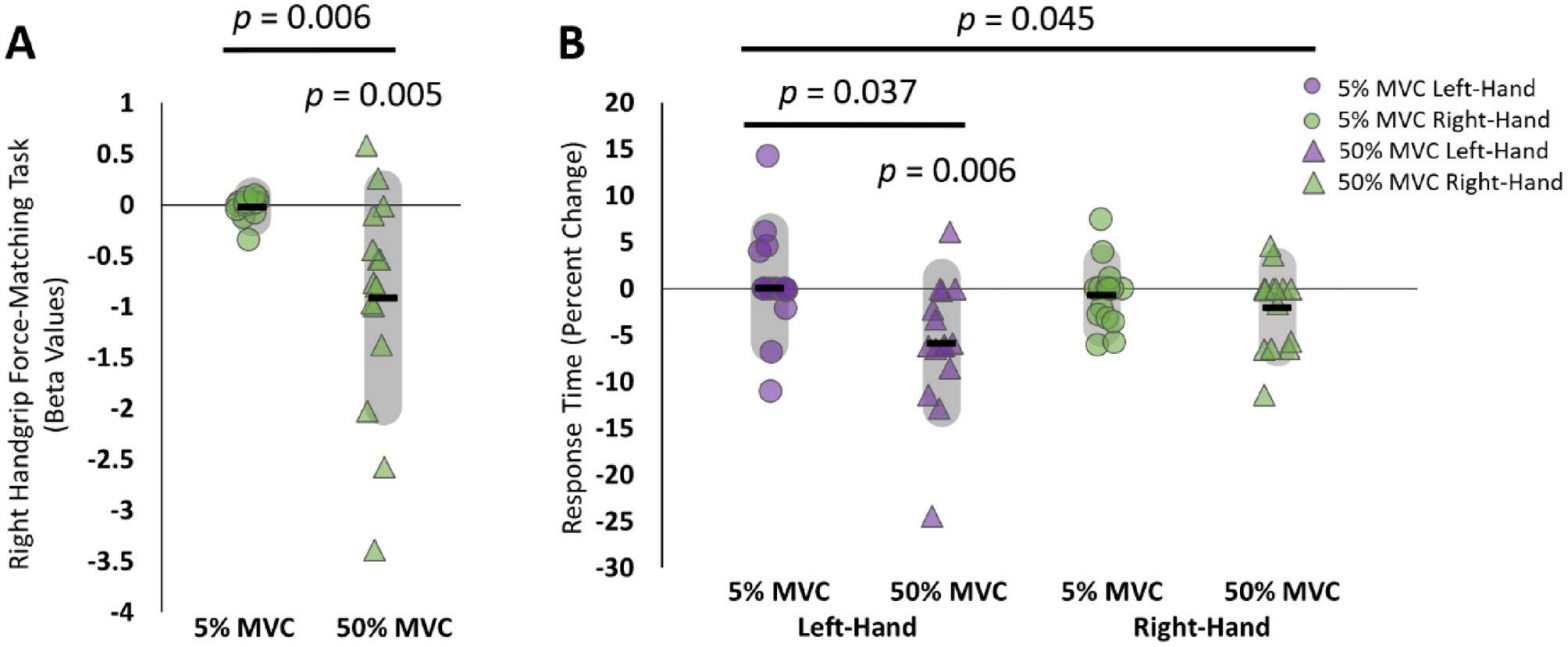
Behavioural changes. (**A**) Beta values for the area under the curve of the force profile from the nine-minute right handgrip force-matching task in experiment one. *p *= 0.005: 50% MVC experienced a significant decline in motor performance (one-sample t-test), *p *= 0.006: a significant difference between conditions (paired-samples t-test), and (**B**) Percent change in response times from pre force-matching task to post force-matching task for the left (purple data points) and right (green data points) hands after the right-hand force-matching task at 5% MVC (circles) and 50% MVC (triangles). *p *= 0.045: significant condition × hand interaction, *p *= 0.037: significant paired sample t-test, *p *= 0.009: significant one-sample t-test change in response times.

We first wanted to determine if the 50% MVC handgrip force-matching task induced a decrease in handgrip force during task performance. We reasoned that the grip force would be reduced over time during our 50% MVC condition, but not during our 5% MVC control condition, consistent with fatigue. We therefore quantified a line of best fit for each participant for each session. In line with our hypothesis, 50% MVC showed a significant decrement in performance over time compared with 5% MVC [50% MVC: β = − 0.912 ± 1.070; 5% MVC: β = − 0.022 ± 0.107; slope of 50% MVC compared with zero: *t*(14) =  − 3.302, *p* = 0.005, g = − 0.824; paired t-test *t*(14) = 3.211, *p* = 0.006, g = 0.801; Fig. 2A].

### Right handgrip contractions increased strength and decreased RTs in the left, contralateral hand

We next investigated whether performance of a 50% MVC force-matching task with the right hand would induce a behavioural improvement of the left hand. For handgrip strength there were missing data due to collection errors that would limit the utility of a RM-ANOVA design. Therefore, we opted to run two separate condition × time linear mixed effects models to assess the pre and post force-matching task MVC data for each hand. For the left hand this analysis revealed a significant main effect of time [*F*(1,13.45) = 22.308, *p* < 0.001] indicating the left hand increased in handgrip force for both conditions (5% MVC: pre = 50.68 ± 26.39, post = 60.89 ± 27.62; 50% MVC: pre = 49.66 ± 20.68, post = 58.99 ± 19.72). However, the condition × time interaction [*F*(1,10.50) = 0.504, *p* = 0.493] and the main effect of condition [*F*(1,6.68) < 0.001, *p* = 0.999] were not significant. For the right hand the non-significant condition × time interaction [*F*(1,23.13) = 0.033, *p* = 0.857], main effect of time [*F*(1,17.37) = 4.010, *p* = 0.061], and condition [*F*(1,9.18) = 0.391, *p* = 0.547] indicate that there were no observable differences in right handgrip force between conditions or across time (5% MVC: pre = 63.29 ± 27.67, post = 58.40 ± 23.67; 50% MVC: pre = 58.41 ± 15.59, post = 55.47 ± 14.75).

For RT data, after running a condition × hand RM-ANOVA with the RT percent change data, we observed a significant condition × hand interaction [*F*(1,14) = 4.84, *p* = 0.045, η_p_^2^ = 0.257], but no significant main effects. To explore the interaction, we ran paired sample t-tests between conditions for each hand separately. This revealed a significant decrease in RTs in the left hand for the 50% MVC condition compared with the 5% MVC condition [5% MVC: pre = 421 ± 60 ms, post = 419 ± 45 ms, Δ = 0.08 ± 5.99%; 50% MVC: pre = 432 ± 45 ms, post = 405 ± 42 ms, Δ = − 5.85 ± 7.05%; *t*(14) = 2.30, *p* = 0.037, g = 0.574]. Further, the left-hand RT improvement was significantly different from zero [*t*(14) =  − 3.21, *p* = 0.006, g = − 0.801]. There were no significant differences for the right hand between the 50% MVC (pre = 409 ± 34 ms; post = 401 ± 42 ms; Δ = − 2.02 ± 4.35%) and 5% MVC (pre = 406 ± 42 ms; post = 403 ± 38 ms; Δ = − 0.73 ± 3.46%) conditions [*t*(14) = 0.788, *p* = 0.441, g = 0.196; Fig. 2B]. For raw response time data see supplementary information.

### Repeated 50% MVC unilateral handgrip contractions increase functional connectivity between the ipsilateral rM1 and right orbitofrontal cortex

**Figure 3:**
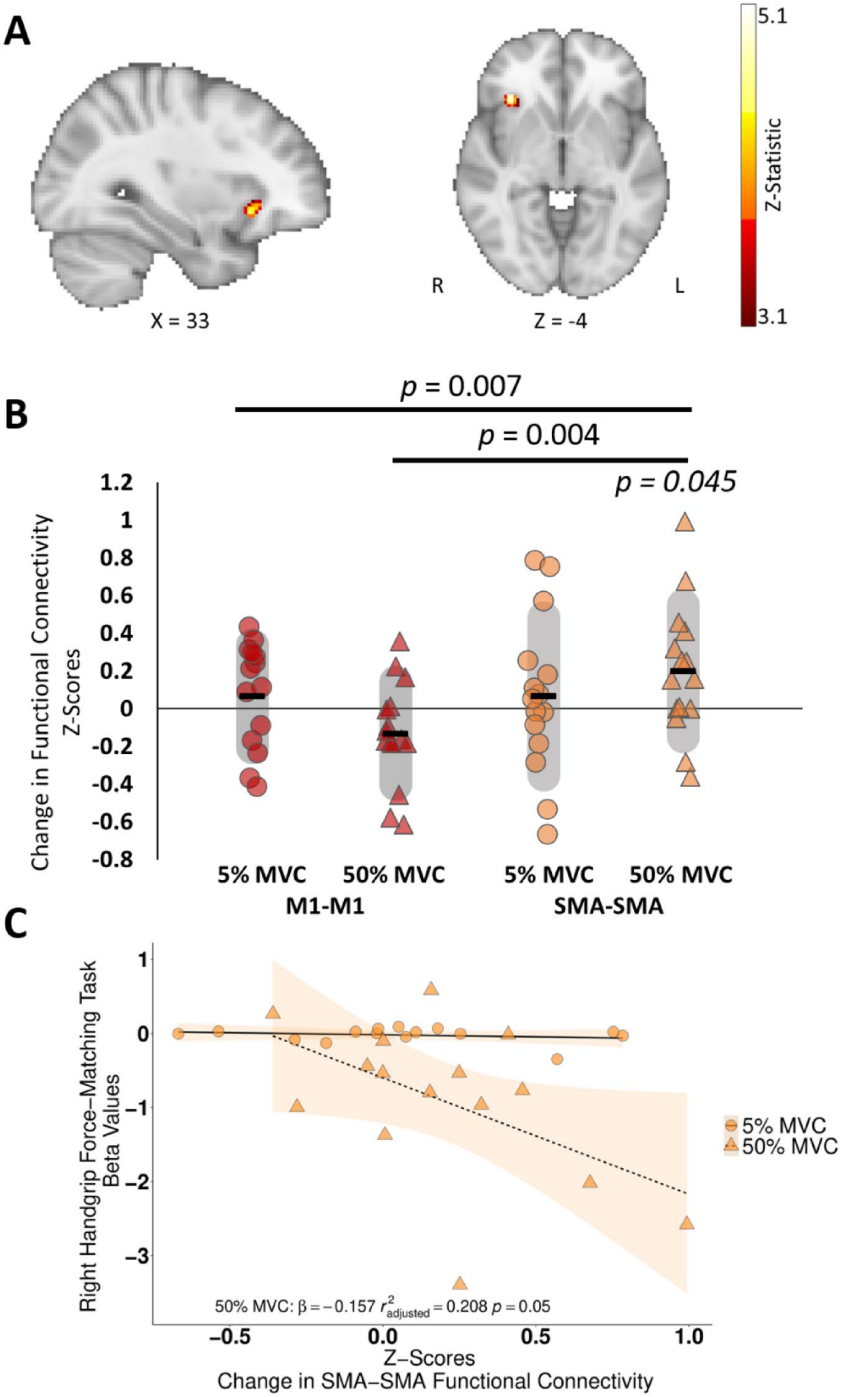
Functional connectivity analyses for (**A**) Seed-based functional connectivity analysis between the right ipsilateral primary motor cortex (seed) and the rest of the brain in the 50% MVC condition, data is a Z-statistic threshold map in standard space. Z-threshold = 3.1. Figure is in radiological view (left on right, right on left). (**B**) Change in interhemispheric functional connectivity between contralateral and ipsilateral primary motor cortices (M1–M1; red data points), and the contralateral and ipsilateral supplementary motor areas (SMA–SMA; orange data points) for 5% MVC (circles) and 50% MVC (triangles). Back horizonal bar represents the mean change. Grey shaded area represents the standard deviation. *p *= 0.007: significant condition × ROI × time interaction, *p *= 0.004: significant ROI × time interaction for 50% MVC condition, *p *= 0.045: significant paired-sample t-test for SMA–SMA connectivity, and (**C**) Scatter plot correlation between X-axis: The change in interhemispheric functional connectivity z-scores between the contralateral left and ipsilateral right supplementary motor areas (SMA-SMA) and Y-axis: beta values representing the decline in motor performance over time (negative slope = performance decline over time) (r^2^_adjusted_ = 0.208, *p *= 0.050, β = − 0.157). Figure 3A was made in MRIcoGL^61^.

To investigate the neural changes associated with our observed behavioural improvements in the left, contralateral hand, we performed a voxel-wise seed-based analysis from the hand area of ipsilateral, rM1. There was a significant increase in functional connectivity with the ipsilateral, right orbitofrontal cortex (rOFC) in the 50% MVC condition, compared with the 5% MVC condition (Table 1; Fig. 3A).

**Table 1:** Post > pre-force-matching task rs-fMRI contrasts from iM1 seed-based functional connectivity analysis.

Voxels	*p*-value	z-max	MNI Coordinates (mm)			Label
			X	Y	Z	
50% MVC						
104	0.0348	5.12	34	26	− 6	Right Orbitofrontal Cortex
5% MVC						
182	0.00187	4.44	28	− 50	− 26	Right Cerebellum VI
104	0.0363	4.78	− 42	− 54	− 4	Left Inferior Temporal Gyrus

### SMA–SMA connectivity increased after 50% MVC contractions

To address our a priori hypothesis that the 50% MVC condition would alter inter-hemispheric functional connectivity between left and right M1, and between left and right SMA, we performed an ROI–ROI connectivity analysis. A RM-ANOVA with one factor of condition (5% MVC, 50% MVC), one factor of ROI (M1, SMA), and one factor of time (pre force-matching task, post force-matching task) revealed a significant three-way (condition × ROI × time) interaction [*F*(1,14) = 10.154, *p* = 0.007, η_p_^2^ = 0.420]; a significant main effect of ROI [*F*(1,14) = 118.113, *p* < 0.001, η_p_^2^ = 0.894], but no significant main effect of condition [*F*(1,14) = 0.508, *p* = 0.488, η_p_^2^ = 0.035], or time [*F*(1,14) = 0.797, *p* = 0.387, η_p_^2^ = 0.054].

To understand this three-way interaction, we then ran separate RM-ANOVA tests for the 50% and 5% MVC conditions, which revealed a significant ROI × time interaction for the 50% MVC condition [*F*(1,14) = 11.970, *p* = 0.004, η_p_^2^ = 0.461] but not for the 5% MVC condition [*F*(1,14) < 0.001, *p* = 0.997, η_p_^2^ < 0.001]. Follow-up tests revealed a significant increase in SMA–SMA connectivity in the 50% MVC condition [*t*(14) =  − 2.203, *p* = 0.045, g = − 0.550, Fig. 3B], but no significant changes in the 5% MVC, nor for the M1 in either condition.

Given the 50% MVC force-matching task led to both a decrease in force output and an increase in SMA–SMA connectivity, we next wanted to investigate whether these two effects were related. We demonstrated a negative correlation between the degree of force decline in the 50% MVC condition and change in SMA–SMA connectivity, such that a greater decline in handgrip performance, indexed by greater decrease in the force output, correlated with an increase in SMA–SMA connectivity (*r*^2^_adjusted_ = 0.208, *p* = 0.050, β = − 0.157; Fig. 3C). This relationship was not observed for M1–M1 connectivity (50% MVC: M1–M1 connectivity and force decline *r*^2^_adjusted_ = − 0.077, *p* = 0.945, β = − 0.076) or in SMA–SMA connectivity in the 5% MVC condition (5% MVC: SMA–SMA connectivity and force decline: *r*^2^_adjusted_ = − 0.024, *p* = 0.427, β = − 0.863).

### Decrease in SMA and M1 GABA correlated with decrease in left-hand RTs after right-hand 50% MVC handgrip contractions

**Figure 4:**
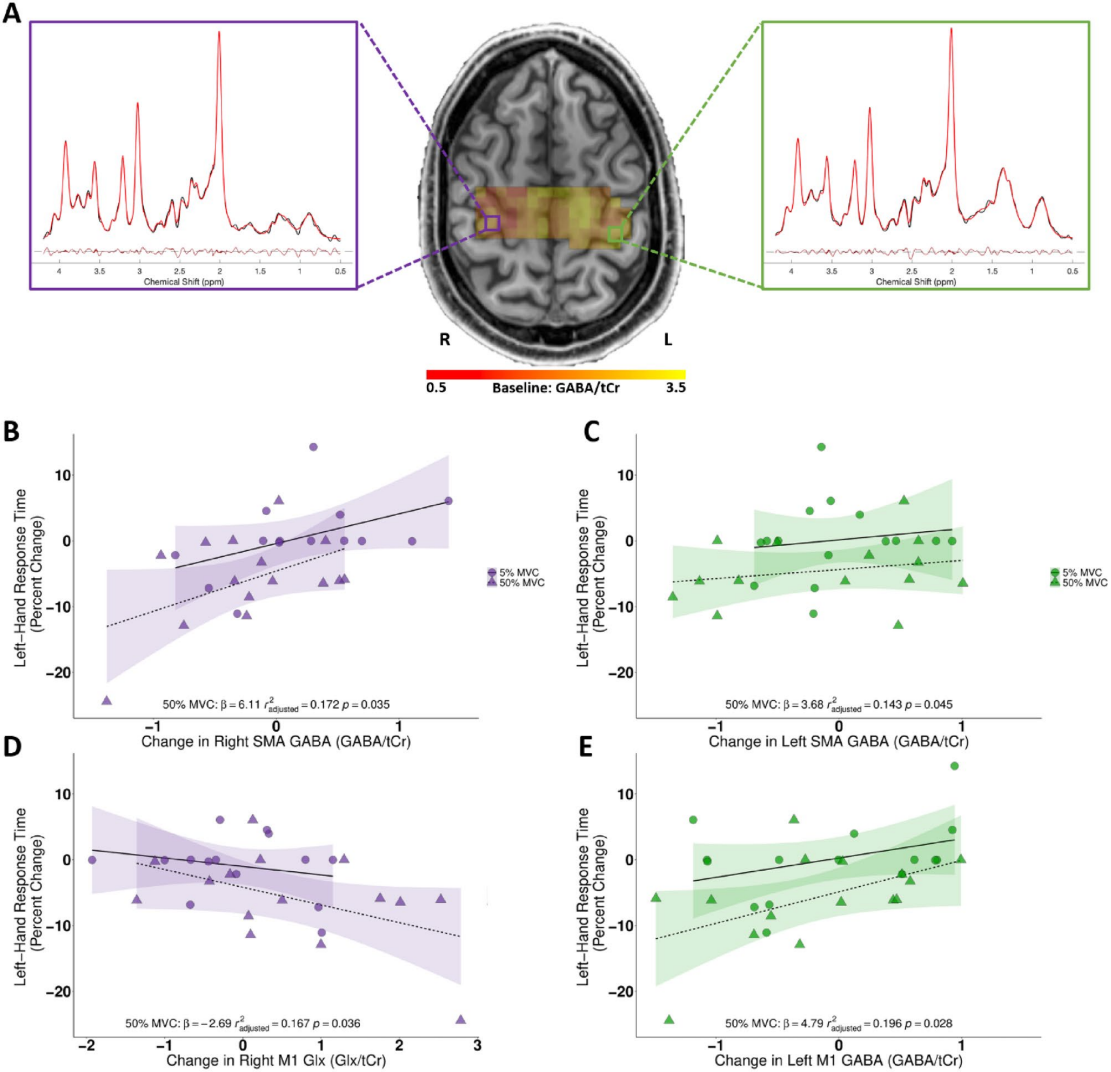
(**A**) Magnetic resonance spectroscopic imaging spectrum from a representative participant, with spectra from M1 voxels in each hemisphere. (**B**–**E**) Scatter plot correlations for the right hemisphere (**B**,**D**; purple) and left hemisphere (**C**,**E**; green) between X-axes: SMA (**B**,**C**; top scatter plots), M1 (**D,E** bottom scatter plots) for GABA/tCr (rSMA, lSMA, lM1) and Glx/tCr (rM1) ratios with Y-axes: the change in left-hand response times (ms) after performing the right-hand force-matching task at either 5% MVC (circles) or 50% MVC (triangles) conditions. Figure 4A was made in fsleyes as part of FSL^39^.

Finally, given our a priori hypothesis that the behavioural effects of unilateral 50% MVC handgrip contractions decrease inhibition, we investigated changes in GABA within each ROI. We ran RM-ANOVA tests for M1 and SMA separately, with one factor of condition (50% MVC, 5% MVC), one factor of hemisphere (left, right) and one factor of time (pre, post). There were no significant main effects or interactions. However, in line with our a priori hypothesis that GABA would decrease and Glx would increase in response to handgrip contractions, there were significant positive correlations between an individual participant’s decrease in RTs for the left-hand and their decrease in lM1 GABA (one-tailed: *r*^*2*^_*adjusted*_ = 0.196, *p* = 0.028, β = 4.79), lSMA GABA (one-tailed: *r*^*2*^_*adjusted*_ = 0.143, *p* = 0.045, β = 3.68), and rSMA GABA (one-tailed: *r*^*2*^_*adjusted*_ = 0.172, *p* = 0.035, β = 6.11) such that the greater the decrease in GABA in these regions, the greater behavioural improvement*.* A significant negative correlation was also observed for the increase in rM1 Glx with the decrease in RTs for the left-hand (one-tailed: *r*^*2*^_*adjusted*_ = 0.167, *p* = 0.036, β = − 2.69; Fig. 4). Importantly, we did not test the relationship between the increase in left handgrip force and the neurochemical changes due to low n in those data.

## Discussion

The objective of this study was to determine the neural correlates of unimanual 50% MVC handgrip contractions and the impact on contralateral limb motor performance. Specifically, we hypothesised that a decrease in handgrip force would be associated with increased sensorimotor functional connectivity^16^ and disinhibition of ipsilateral motor areas^13^, neural effects that lead to enhanced performance of the contralateral, non-contracting hand.

We showed that right handgrip contractions at 50% MVC, but not 5% MVC, resulted in a decline in right handgrip force, something that would be consistent with fatigue, though other interpretations are possible. We also observed, in line with our hypothesis, that repeated 50% MVC handgrip contractions performed with the right-hand led to significant improvements in left hand MVCs and RTs, something that was not seen either in the right hand, nor for the low force 5% MVC condition (where only left MVC improved). These improvements were also accompanied by neural changes. We observed a significant interhemispheric interaction between a decline in M1–M1 connectivity and an increase in SMA–SMA connectivity. The increase in SMA–SMA connectivity correlated with the degree of force decline on a participant-by-participant basis. Additionally, increased rM1–rOFC connectivity was observed on a voxel-wise analysis. Finally, we used MRSI to quantify potential changes in GABA and Glx. We observed no group mean changes, but on a participant-by-participant basis, GABA decreases in the lM1 and bilateral SMAs modestly correlated with behavioural improvements.

### Increased functional connectivity between ipsilateral rM1 and rOFC after repeated right 50% MVC handgrip contractions

We observed increased functional connectivity between our rM1 seed and the rOFC after right 50% MVC handgrip contractions. Increased OFC activity has been associated with faster response times^62^ and hand motor learning^63^. The increased rM1–rOFC functional connectivity observed here may therefore reflect the OFC’s role as a top-down motor control region^64^ which is involved in the regulation of motor responses and error monitoring^63^. In line with this hypothesis, Jackson et al.^65^ showed increased activation in the right OFC in participants who significantly improved their performance in a right-hand motor task, either having physically executed the task or having performed motor imagery. Taken together, our results suggest that increased functional connectivity between the rM1 and the rOFC may be functionally relevant for improved RTs in the contralateral, non-contracting left-hand.

### Repetitive 50% MVC contractions increase SMA-SMA connectivity

Our ROI-based functional connectivity analysis examined how the temporal correlations between left and right M1 and SMA homologs changed after the right handgrip force-matching task. After 50% MVC contractions, SMA-SMA functional connectivity increased, and the degree of change was negatively correlated with the degree of decreased handgrip force, as indexed by a decrease in the area-under-the-curve for each contraction over time, such that participants who exhibited greater declines in this metric showed greater increase in SMA–SMA functional connectivity.

The modulation of the ipsilateral SMA after unilateral fatigue may be explained in part due to its dense interhemispheric homologous connections^66^ and is thought to play an important role in interhemispheric communication in the movement preparation phase^67^. The inter-connectedness of the SMA between hemispheres offers insight as to why unilateral fatiguing exercise modulates the ipsilateral motor network. Further, lesion studies have demonstrated that impairments to the SMA cause increased response time (i.e., slower movement)^68^.

The SMA has been described as a ‘phylogenetically older’ M1^69^ which has direct excitatory connections not only onto the M1 circuitry^70,71^, but onto alpha-motoneurons that innervate the hand and fingers^72,73^. The SMA plays a major role in the planning and self-initiation of voluntary movement, and serves to integrate multimodal information to ensure motor and visual systems are in agreement^74,75^. The increased connectivity between left and right SMA in the present study may reflect a mechanism by which repetitive unilateral 50% MVC handgrip contractions induced neural changes that improve motor performance for the inactive, contralateral hand. Therefore, it is plausible that the 50% MVC condition-specific increase in SMA–SMA functional connectivity improved contralateral left-hand RTs through direct corticospinal connections to upper limb alpha-motoneurons via the cortico-reticulospinal tract^26^. Further, transcallosal homologous connectivity between motor regions primarily reflects inhibitory processes^76^. Therefore, an increase in SMA–SMA functional connectivity may reflect an increase in interhemispheric inhibition between these premotor regions. Given this, we can hypothesize that an increase in interhemispheric SMA–SMA functional connectivity, reflecting interhemispheric inhibition, may aid in silencing unwanted cortical activity that facilitates a more focal excitatory process in the opposite hemisphere^77^ giving rise to enhanced motor performance (i.e., faster RTs) in the opposite hand.

### A decrease in lM1 and bilateral SMA GABA after a decrease in handgrip force correlates with improvements in left-hand RTs

To address our hypothesis that the 50% MVC contractions would lead to decreased inhibition in motor regions, we used a novel MRSI sequence to quantify neurochemical concentrations across the sensorimotor network. At a group level, there were no significant GABA changes in M1 and SMA in either hemisphere after 50% MVC contractions compared with 5% MVC control condition. However, in line with our a priori hypothesis, we did observe significant one-tailed, uncorrected correlations on a participant-by-participant basis between the decrease in lM1, lSMA, and rSMA GABA after right 50% MVC contractions and the improved behaviour in the non-contracting hand. While the mechanism by which lM1 and bilateral SMA GABA decreases lead to behavioural improvement in the left contralateral hand after right handgrip contractions is unclear, a combination of decussated corticospinal tract and non-decussated corticospinal or cortico-reticulospinal tract pathways may play an important role in modulating left hand motor output^12,14^. For example, the cortico-reticulospinal tract is known to have descending connections from the SMA that synapse onto motor neuronal pools that innervate the proximal and distal segments of the upper limb^20–24^, and this pathway contributes to reductions in RTs^24^ and also neuromuscular strength^22,25^.

### Future directions

The present work investigated the effect of repetitive 50% MVC right handgrip contractions on motor performance with the left hand in right-handed individuals. It is presently unclear whether this effect would persist if the experiment were repeated in left-handed participants, or if the effect would also be present if the experiment were reversed with the handgrip contractions performed in the left hand and motor performance assessed in the right. Future research should also investigate the specificity of the effect in terms of task type and complexity. In this work we showed improved response times and handgrip contraction strength, but this work does not shed light on whether the performance enhancement would be present with more complex motor learning or cognitive-motor tasks. These are important experimental paradigms that should be assessed in the future to better appreciate the context in which unilateral handgrip fatigue may be useful in promoting contralateral limb motor behaviour.

## Conclusions

This study identified that repetitive 50% MVC right handgrip contractions led to behavioural improvements in the opposite hand which were accompanied by increased SMA–SMA functional connectivity. In addition, we demonstrated an increase in functional connectivity between rM1 and the rOFC. We also demonstrated positive relationships between change in inhibition (GABA) in lM1 and bilateral SMA and a negative relationship between the change in rM1 Glx with the behavioural improvement of RTs in the non-contracting left-hand. These results suggest that repetitive 50% MVC unimanual contractions can enhance subsequent motor performance of the opposite hand. This approach may serve as a promising adjunct therapy to prime the ipsilateral sensorimotor system for rehabilitation in a range of neurological or orthopedic conditions.

## Supplementary Information

Supplementary Information.

## Data Availability

All data will be available from the authors upon reasonable request.
