# Supplementary material for: Repeated unilateral handgrip contractions alter functional connectivity and improve contralateral limb response times
Source: Sci Rep. 2023 Apr 20;13:6437. doi: 10.1038/s41598-023-33106-1 (PMC10119116; doi:10.1038/s41598-023-33106-1)
Supplement: Supplementary file 1 — Supplementary Information. [file 41598_2023_33106_MOESM1_ESM.docx]

## Motion parameters during resting-state fMRI data

Participants were able to successfully remain still during all resting-state scans, as evident by the low relative mean displacements metrics estimated by MCFLIRT. Overall, across both conditions and pre and post force-matching task MRI scans there was a low relative mean displacement (0.05 ± 0.21 mm, table 2).

| **Supplementary Table 1.** Relative mean displacements in mm | | | | | | |
| --- | --- | --- | --- | --- | --- | --- |
|  | **5% MVC** | | **50% MVC** | | |  |
| **Subject** | **Pre** | **Post** | | **Pre** | **Post** |  |
| sub-001 | 0.05 | 0.05 | | 0.06 | 0.06 |  |
| sub-002 | 0.06 | 0.05 | | 0.06 | 0.05 |  |
| sub-003 | 0.04 | 0.04 | | 0.05 | 0.04 |  |
| sub-004 | 0.06 | 0.05 | | 0.06 | 0.05 |  |
| sub-005 | 0.05 | 0.07 | | 0.05 | 0.15 |  |
| sub-006 | 0.04 | 0.04 | | 0.05 | 0.05 |  |
| sub-007 | 0.04 | 0.04 | | 0.05 | 0.04 |  |
| sub-008 | 0.08 | 0.08 | | 0.06 | 0.07 |  |
| sub-009 | 0.03 | 0.04 | | 0.03 | 0.04 |  |
| sub-010 | 0.09 | 0.08 | | 0.08 | 0.12 |  |
| sub-012 | 0.06 | 0.06 | | 0.08 | 0.07 |  |
| sub-013 | 0.04 | 0.04 | | 0.03 | 0.04 |  |
| sub-014 | 0.08 | 0.08 | | 0.09 | 0.08 |  |
| sub-016 | 0.05 | 0.06 | | 0.06 | 0.06 |  |
| sub-017 | 0.06 | 0.05 | | 0.06 | 0.06 |  |

----

| **Supplementary Table 2.** Response times in milliseconds | | | | | | | | |
| --- | --- | --- | --- | --- | --- | --- | --- | --- |
|  | **5% MVC** | | | | **50% MVC** | | | |
|  | **Left hand** | | **Right hand** | | **Left hand** | | **Right hand** | |
| **Subject** | **Pre** | **Post** | **Pre** | **Post** | **Pre** | **Post** | **Pre** | **Post** |
| sub-001 | 384.49 | 384.45 | 384.04 | 376.20 | 384.57 | 383.62 | 359.93 | 359.82 |
| sub-002 | 459.37 | 459.23 | 446.82 | 434.50 | 484.37 | 473.64 | 467.70 | 484.39 |
| sub-003 | 444.07 | 434.44 | 409.76 | 384.76 | 384.79 | 384.78 | 384.80 | 384.73 |
| sub-004 | 384.75 | 384.69 | 384.87 | 384.68 | 434.51 | 397.26 | 384.80 | 384.67 |
| sub-005 | 400.70 | 416.64 | 384.67 | 384.80 | 463.02 | 434.51 | 434.51 | 434.52 |
| sub-006 | 359.67 | 358.74 | 335.11 | 359.96 | 384.66 | 335.04 | 384.60 | 359.67 |
| sub-007 | 384.79 | 384.57 | 384.59 | 384.38 | 435.04 | 409.49 | 384.87 | 359.74 |
| sub-008 | 335.06 | 382.88 | 384.53 | 384.70 | 384.62 | 359.83 | 384.63 | 359.76 |
| sub-009 | 384.65 | 384.69 | 384.93 | 384.84 | 409.52 | 384.69 | 434.21 | 409.56 |
| sub-010 | 488.64 | 434.52 | 441.96 | 447.11 | 409.52 | 434.35 | 409.64 | 409.49 |
| sub-012 | 466.42 | 434.56 | 434.43 | 409.51 | 434.54 | 384.94 | 384.78 | 384.40 |
| sub-013 | 463.04 | 484.10 | 442.08 | 459.20 | 462.99 | 434.49 | 441.78 | 434.53 |
| sub-014 | 409.46 | 434.33 | 384.63 | 384.57 | 384.55 | 384.62 | 384.45 | 384.51 |
| sub-016 | 384.75 | 384.77 | 384.08 | 371.83 | 509.29 | 384.84 | 434.55 | 384.71 |
| sub-017 | 566.08 | 525.36 | 509.36 | 491.34 | 509.28 | 492.53 | 463.11 | 484.30 |
